# Communicating Hydrocephalus Following Eosinophilic Meningitis Is Pathogenic for Chronic Viliuisk Encephalomyelitis in Northeastern Siberia

**DOI:** 10.1371/journal.pone.0084670

**Published:** 2014-02-28

**Authors:** Alexander Storch, Jan Kassubek, Hayrettin Tumani, Vsevolod A. Vladimirtsev, Andreas Hermann, Vladimir L. Osakovsky, Vladimir A. Baranov, Vadim G. Krivoshapkin, Albert C. Ludolph

**Affiliations:** 1 Division of Neurodegenerative Diseases, Department of Neurology, Dresden University of Technology, Dresden, Germany; 2 German Center for Neurodegenerative Diseases (DZNE) Dresden, Dresden, Germany; 3 Department of Neurology, University of Ulm, Ulm, Germany; 4 North-Eastern Federal University (NEFU), Scientific Research Institute of Health, Yakutsk, Republic of Yakutia, Russian Federation; 5 Department of Radiology, National Medical Center of Yakutia, Yakutsk, Republic of Yakutia, Russian Federation; Friedrich-Alexander University Erlangen, Germany

## Abstract

**Background:**

Viliuisk encephalomyelitis (VE) is an endemic neurological disease in Northeast Siberia and generally considered to be a chronic encephalomyelitis of unknown origin actually spreading in the Sakha (Yakutian) Republic.

**Methodology and Principle Findings:**

In search for the pathophysiology and causative agent of VE, we performed a cross-sectional study on clinical, serological and neuroimaging data on chronic VE patients during two medical expeditions to three villages within the Viliuiski river basin in the Republic of Sakha in 2000 and to the capital Yakutsk in 2006. The severity of the core clinical picture with predominant sensory ataxia, gait apraxia, lower limb spasticity, cognitive impairment and bladder dysfunction correlated with the degree of MRI findings showing enlargement of inner ventricular spaces as in communicating hydrocephalus. Laboratory studies revealed transient eosinophilia during the preceding acute meningitis-like phase, but no ongoing inflammatory process in the CSF. We found immune reactions against *Toxocara canis* in the majority of chronic VE patients but rarely in controls (*P* = 0.025; Fisher's exact test). Histological analysis of subacute to subchronic VE brain samples showed eosinophilic infiltrations with no signs of persistent *Toxocara canis* infection.

**Conclusions and Significance:**

Our data showed that pressure by the communicating hydrocephalus as a mechanical factor is the major pathogenic mechanism in chronic VE, most likely triggered by eosinophilic meningitis. There are no signs for an ongoing inflammatory process in chronic VE. The past eosinophilic reaction in VE might be caused by *Toxocara* ssp. infection and might therefore represent the first hint for an initial cause leading to the development of chronic VE. Our data provide a framework for future studies and potential therapeutic interventions for this enigmatic epidemic neurological disease potentially spreading in Sakha Republic.

## Introduction

**Figure 1:**
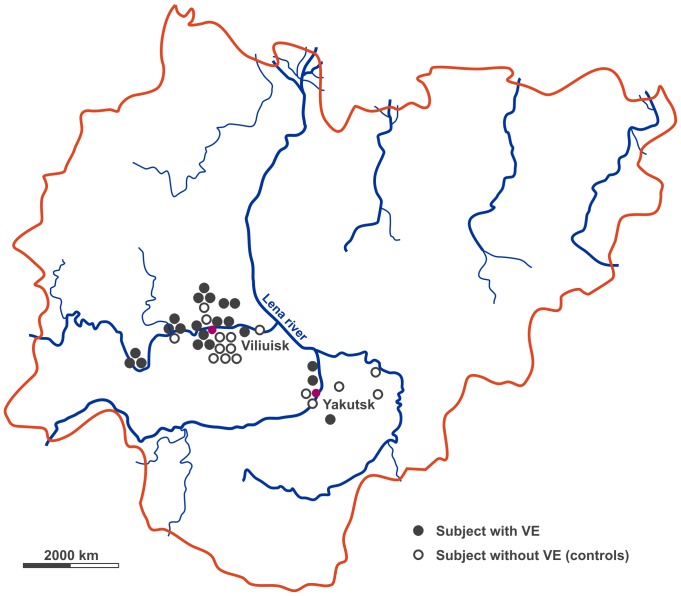
Schematic map of the Republic of Sakha marking the places of origin of studies subjects. Closed symbols represent chronic VE patients, open symbols indicate controls (refer to text for details), red symbols mark cities/villages.

Viliuisk encephalomyelitis (VE) was first described in 1887 by the German Richard Maak in a rural population left of the Viliuisk river within Sakha Republic (Yakutia) within Northeast Siberia [1], [2]. VE is an endemic neurological disease in the Viliuiski river basin and the surrounding rural areas including the region of the capital Yakutsk (Figure 1), similar to other neurological conditions within geographical isolates such as kuru or the ALS-PD-dementia complex of Guam. The disease is locally named “bokhoror”, meaning stiffness of the legs. Goldfarb and Gajdusek pioneered studies on the phenotype in 1992 and suggested that VE consisted of an acute encephalitic phase followed by a chronic infection provoking an extensive inflammatory response with spongiform regions of the central nervous system [3]–[5]. The authors reported the clinical picture as meningitis-like in the acute phase, and as a progressive, sometimes self-limited syndrome in the chronic phase consisting of spastic tetraparesis with gait disturbances, bradykinesia, rigidity, incontinence, and dementia [3], [4], [6]. These data led to the hypothesis of a chronic spongiform infectious process as the major cause of VE [3], but this hypothesis could never be proven in spite of intensive efforts. Recent preliminary data from cerebrospinal fluid (CSF) studies pointed to an unknown herpes or herpes-like virus as a causative agent, but PCR analysis failed to detect herpes virus genome in VE [2]. The recent tremendous efforts in VE research in an isolated remote area of the world without advanced medical technologies available [2], [5], [7], [8] were not able to define or even hypothesize on the pathophysiological events leading to chronic VE. Although the number of VE cases seems to decline in recent years, VE appears to be spreading in Sakha Republic and thus the impact of VE on global public health is not predictable [2], [5], [7].

We performed a cross-sectional study on clinical, serological and neuroimaging data on chronic VE patients during two medical expeditions to the Republic of Sakha to gain hypothesis on the pathophysiology and causative agent of chronic VE.

## Methods

### Recruitment and subjects

During two medical expeditions to three villages within the Viliuiski river basin in the Republic of Sakha in 2000 and to the capital Yakutsk in 2006 (**Figure 1**), we focused on a small group of chronic VE patients with precise phenotype definition, a pivotal prerequisite for defining underlying pathophysiological processes in complex disorders [9]. We thus selected 20 patients (54.1%) with chronic VE (median age: 55 [range 20–71] years, sex: 17 males/3 females) out of 37 patients previously assigned to suffering from VE (see **Table 1** for details on patient cohorts; see **Table S1** for STrengthening the Reporting of OBservational studies in Epidemiology [STROBE] initiative checklist). The other 17 patients were found to be neurologically normal at the time of examination (n = 9), two patients met criteria for subcortical vascular encephalopathy (SVE; 11.8%), one for autosomal-dominant hereditary spastic paraplegia (HSP; 5.9%), one for multiple system atrophy (MSA-P; 5.9%), one for autosomal-recessive chorea-acanthocytosis (5.9%), one for multiple sclerosis (MS; 5.9%), one for dominant hemiplegic migraine (5.9%), and one patient showed S1 radiculopathy. We point to the fact that patient recruitment, clinical investigations and biosample collections were performed in an isolated, rural and remote region of Northeastern Siberia with only limited access to advanced medical care, which might explain the low percentage of patients with the core syndrome of chronic VE within the original 37-patients cohort.

**Table 1:** Demographic characteristics of patient cohorts.

	All patients previously assigned to VE	Chronic VE	Chronic VE (CSF/serostudies and neuroimaging subcohort)	Chronic VE (retrospective neuroimaging cohort)
N	37	20	10	12
**Demography**				
Female/male	8 (22%)/29 (78%)	3 (15%)/17 (85%)	1 (10%)/9 (90%)	3 (25%)/9 (75%)
Age (years, mean±SD [range])	49.2±13.4 (17 to 71)	51.9±14.0 (20 to 71)	56.1±9.0 (44 to 71)	50.0±9.0 (29 to 64)
Disease duration (years, mean±SD [range])	n.a.	25.7±11.9 (5 to 51)	26.5±7.7 (19 to 40)	20.0±9.0 (2 to 34)
**Clinical symptoms**				
Acute onset (n [%])#	n.a.	12 of 18 (67%)	6 of 10 (60%)	10 of 12 (83%)
with cranial nerve deficits (n [%])#	n.a.	6 of 8 (75%)	3 of 9 (33%)	n.d.
Progressive course (n [%])#	n.a.	14 of 16 (88%)	10 of 10 (100%)	9 of 11 (82%)
Gait apraxia (n [%])	n.a.	19 of 20 (95%)	9 of 10 (90%)	11 of 12 (92%).
Sensory ataxia (n [%])	n.a.	20 of 20 (100%)	10 of 10 (100%)	n.d.
Spasticity of lower limbs (n [%])	n.a.	18 of 20 (90%)	9 of 10 (90%)	n.d.
Urge incontinence (n [%])	n.a.	14 of 20 (70%)	9 of 10 (90%)	n.d.
Dysarthria (n [%])	n.a.	16 of 20 (80%)	8 of 10 (80%)	n.d.
Dementia+	n.a.	8 of 14 (57%)	4 of 7 (57%)	n.d.

Data are number of patients (%) or median (range), unless otherwise stated. n.a., not applicable; n.d., no data available.

The VE patient CSF cohort and the Yakutian control CSF cohort did not statistically differ with respect to gender distribution (*P* value of 0.396; Pearson's Chi-Square test) or age (*P* value of 0.234; Student's t-test).

# Data from medical history (not obtainable from all patients).

+ Detailed assessment of neuropsychological deficits was not possible because of the language barrier, but we were able to obtain data on cognitive function together with our translators in 14 of the 20 VE patients.

We obtained medical history and clinical data from all VE patients, electrophysiology data from 10, CSF analyses from 10, and magnetic resonance imaging (MRI) of head and upper spinal cord in 10 patients. It is noted that we were not able to obtain detailed medical history data from all patients due to limited case record file archiving.

### Ethics statement

All patients or next of kin provided written informed consent to V.A.V., V.L.O. or V.G.K. and the study and documentation processes were approved by institutional review board of the Institute of Viliuisk Encephalomyelitis at the Academy of Science of the Republic of Sakha (Yakutia), renamed as North-Eastern Federal University (NEFU), Scientific Research Institute of Health.

*Toxocara (T.) canis* infected mouse brain tissue was kindly provided *post-mortem* from by the Centre for Infectious Disease Control Netherlands, National Institute of Public Health and the Environment (RIVM), Bilthoven, The Netherlands. All animal experiments were performed as described previously and according to the International and Institutional Guidelines for Animal Care (Committee on Animal Experimentation of the RIVM) [10].

### Clinical electrophysiology

We obtained standard electrophysiology data (neurography of the ulnar, median, tibial and peroneal nerve, myography of the M. tibialis anterior, and somato-sensory evoked potentials by stimulating the medial and tibial nerves on both sides) from 10 VE patients and five Yakutsian controls using the portable Neuromax 1002 apparatus (MTS Medizintechnik GmbH, Munich, Germany). Somato-sensory evoked potential studies revealed no reliable potentials in five patients due to technical reasons.

### Cerebrospinal fluid (CSF) studies

Cerebrospinal fluid (CSF) samples were collected from a total of 10 VE patients and 8 control individuals (female/male: 2 (25%)/6 (75%); mean±SD age: 45.5±14.0 years (range: 17 to 67 years); 3 healthy controls, 5 disease controls [2 patients with SVE, one with MS, one with HSP, one with familiar hemiplegic migraine]; see **Table 1** for details on patient cohorts). We used standard non-traumatic Sprotte needle systems for obtaining the CSF samples, which were further processed in the Laboratory of Clinical Chemistry at the Institute of VE, Yakutsk (by V.L.O. and H.T.). CSF cell counts were visually determined in a Fuchs-Rosenthal chamber within 30 minutes after lumbar puncture. Following leukocyte count, native CSF was processed for differential cytology using the Sayk chamber. Staining was performed according to Pappenheim protocol. Native CSF was centrifuged within 15 minutes after lumbar puncture, and supernatant samples were aliquoted into 1 ml screw tubes and stored at −70°C until further analysis at the CSF laboratory of the Dept. of Neurology, University of Ulm, Germany (by H.T.).

Routine CSF analysis included leukocyte count, differential cell count, CSF lactate, CSF total protein, albumin CSF/serum quotient, IgG-, IgA and IgM quotients, oligoclonal IgG bands. The concentrations of albumin, IgG, IgA, and IgM in supernatant CSF and in serum were measured using a Behring Nephelometer Analyser (Prospec, Dade Behring, Marburg, Germany) using a polyclonal antibody (in case of albumin, IgG and Beta-Trace) or a latex-enhanced antibody based reaction (in case of IgA and IgM). Detection of oligoclonal IgG bands was performed by isoelectric focusing on agarose gel and subsequent immunoblotting using IgG specific antibody staining.

### Immunodiagnostics

The extended CSF analysis comprised serological studies against several viruses for calculation of the antibody index (AI) for Measles, Rubella, Varicella Zoster, Herpes simplex 1 and 2, CMV, and FSME (tick borne encephalitis) virus using commercially available enzyme immunoassays (Virotech, Germany). Further serological studies included the AI for *Borrelia burgdorferi* and *Treponema pallidum* antibodies. AI values were calculated from the arbitrary concentrations in CSF and serum according to the formula of Reiber and Felgenhauer [11]. Intrathecal synthesis was assumed in case of antibody index larger than 1.4. Serum and CSF samples were sent to the Bernhard-Nocht-Institute for Tropical Medicine in Hamburg, Germany, for lymphocytic choriomeningitis virus (LCMV) diagnostics using an immunofluorescence assay (IFA) essentially as described by Ceianu and colleagues [12].

We then measured organism-specific antibodies against the nematode species *Toxocara canis* in CSF and sera using immunoblotting. *Toxocara* antibodies were detected using lyophilized *T. canis* excretory/secretory larval antigens from Bordier Affinity Products SA (Crissier, Switzerland) and standard nitrocellulose membrane immunoblot methods (secondary antibody: rabbit anti-human IgG marked with HRP from DAKO, Hamburg, Germany). Blots with the typical 7-band patterns were judged as positive [13]. *Angiostrongylus cantonensis* immunoblots were performed in serum on a commercial basis by the Diagnostic Centre, Swiss Tropical Institute in Basel, Switzerland, according to Bärtschi and wo-workers [14].

Antinuclear antibodies were analyzed using an immunofluorescence assay to assess the presence of an autoimmune process.

### Neuroimaging studies

During the 2006 expedition, we performed magnetic resonance imaging (MRI) of the brain and upper spinal cord and acquired data in 10 chronic VE patients and 4 Yakutsian controls [female/male: 0 (0%)/4 (100%); mean±SD age: 33.0±13.0 years (range: 18 to 41 years)]. The MRI data sets were acquired on a clinical scanner in the Department of Radiology, National Medical Center of Yakutia, 677000 Yakutsk, Republic of Sakha (Yakutia). The MRI scanner was a Magnetom Impact/Expert scanner (Siemens, Erlangen, Germany), equipped with standard head or spine coils, operating at 1.0 Tesla. The acquisition protocol which was standardized for all 14 subjects consisted of about 10 different sequences (sometimes varying due to subjects' individual needs to shorten the protocol). The sequences included for head/brain a double echo: proton-density (PD) w and T2 w transaxial scan (TSE), T2 w sagittal scan (TSE), T1 w coronar scan (SE), T1 w transaxial scan (SE), (fluid attenuated) inversion recovery transaxial scan (TIRM), inversion recovery coronar scan (TIRM), sometimes T1 w sagittal scan (TSE), and for the spine a T1 w sagittal scan of the cervical myelon (TSE), T2 w sagittal scan of the cervical myelon (TSE), inversion recovery sagittal scan of the cervical myelon (TIRM), T1 w sagittal scan of the thoracic myelon (TSE), T2 w sagittal scan of the thoracic myelon (TSE), in cases of difficulties of assessment additional transaxial T2 w scans. Only in few cases, contrast medium (Magnevist) was administered (n = 2).

Since no 3D T1 datasets could be acquired due to scanner limitations, we estimated the volume of the lateral ventricles, the third ventricle and the total ventricular volume by planar measurement of ventricular area on all slices containing ventricular spaces using the *MRreg* software [15]. To normalize for head size, we calculated the ratio of the ventricular area to the respective intracranial area in each slice (see **Figure S1**). The individual index was calculated by summing up the ratios of all single slices. We compared the data obtained in VE patients (mild VE patients defined to be able to walk without assistance [n = 4], severe VE patients defined to be unable to walk or need assistance [n = 6]), with healthy Yakutian controls (n = 4;) as well as age and sex-matched Caucasian controls [female/male: 1 (7%)/13 (93%); mean±SD age: 50.0±12.0 years (range: 23 to 69 years)] scanned at the Department of Neurology, University of Ulm, Germany, using a 1.5T scanner (Magnetom Symphony, Siemens, Erlangen, Germany) and the same analysis tool to estimate ventricular volume. We included this second control group, because further MRI scanning of healthy Yakutian probands were restricted due to public health reasons (the scanner used is the only scanner in the Republic of Sakha and therefore urgently needed for taking care of other patients).

We were able to analyze additional 12 cranial MRI or computed tomography (CT) scans from chronic VE patients previously collected by the VE Institute of the Academy of Sciences of Yakutia, Yakutsk, between 1995 and 2000 (see **Table 1** for demographic information).

### Histology

We were able to investigate samples of paraffin embedded formalin fixed brain tissue from 3 VE patients from the series published previously by McLean and colleagues [4] (retrospective analyses of anonymous samples were performed according to §15 of the Saxonian Medical Association's professional code of conduct). These patients died before 2000 and were thus not part of our clinical cohorts and only limited clinical data from these patients are available. According to these data, the tissue samples were from patients who died in the subacute to subchronic VE phase [4]. Paraffin specimens were cut in 20 µm slices and deparaffinized and hydrated using routine protocols. Tissue sections were stained with Haematoxylin & Eosin as well as with Giemsa.

For immunofluorescence stainings, tissue sections were pre-incubated in 3% blocking serum containing 0.2% Triton X-100 in PBS for 2 hrs at room temperature and then incubated 12–72 h at 4°C with primary antibodies followed by secondary fluorescence conjugated antibodies for 1–4 h at room temperature. Cell nuclei were counterstained with 4,6-diamidino-2-phenylindole (DAPI). For DAB staining slides were incubated in 3% H_2_O_2_ (Sigma Aldrich, St. Louis, MO) for 30 min at room temperature followed by pre-incubated in 3% blocking serum containing 0.2% Triton X-100 in PBS for 2 hrs at room temperature and then incubated 72 h at 4°C with primary antibodies. Biotinylated secondary antibody (Jackson, West Grove, PA) was used (4 hrs at room temperature) and finally avidin-biotinhorseradish peroxidase complex (Vectastain ABC Elite kit) for 60 min at room temperature. The reaction product was developed with the DAB (Sigma). Sections were dehydrated in ascending alcohol concentrations and Xylol and mounted in Neomount medium. Stained slides were analyzed with a Zeiss inverted microscope (Zeiss Axiovert 35) or Leica fluorescence microscope (Leica DM IRE2, Wetzlar, Germany). Rabbit polyclonal anti-Tcan antibody was kindly provided by Prof. Herbert Auer (Institute of Specific Prophylaxis and Tropical Medicine, Medical University Vienna) and used 1∶500–1∶4000. Rabbit anti-ECP was used 1∶500. It is noted that only these three old tissue samples of limited quality are available and thus no further extensive immunohistochemistry studies are possible.

As a positive control for eosinophilia, we used paraffin embedded blinded specimen suffering from confirmed histiocytosis, kindly provided by the Institute of Pathology at the Dresden University of Technology (written informed consent was obtained according to local hospital care regulations). For *T. canis* stainings, we obtained frozen brains from mice which received *T. canis* egg inoculation 90 days prior to investigation (kindly provided by Dr. Jereon Roelfsema). We analyzed brains of mice infected with *T. canis* as well as respective control mice.

### Statistics

Statistical analyses were performed with the SPSS 18.0 software for Microsoft Windows (SPSS, Chicago, IL). Analysis of normality was performed with the graphical method of normal probability-quantile plot in combination with the Kolmogorov-Smirnov test. Differences between groups in demographical data were evaluated using ANOVA model and Pearson's Chi-Square test as appropriate. Differences of ventricular area/volume between Caucasian controls, Yakutian controls, mild VE and severe VE were assessed using an analysis of variance (ANOVA) model with *post-hoc t*-test and Bonferroni correction. Differences of other parameters (serological results) between VE patients and (Yakutian) controls were assessed by Pearson's Chi-Square test or Fisher's exact test (with Bonferroni corrections in *post-hoc* tests), Mann-Whitney U test or Student's t-test as appropriate. Multiple logistic regression analysis was used to assess the association of co-variables (age, gender, duration of disease) with ventricular area/volume. Since no co-variable had predictive value univariate analyses were performed. Data are presented as mean ± standard deviation (SD) and range. Values of *P*<0.05 were considered statistically significant.

## Results

Chronic VE patients displayed a homogenous clinical phenotype consisting of sensory ataxia (100% of all VE patients), gait apraxia (95% of all patients), spasticity predominantly of the lower limbs (90%), dysarthria (80%), and urge incontinence (70%). Detailed assessment of neuropsychological deficits was not possible because of the language barrier, but according to the individual histories available in 14 patients, we were convinced that 57% of VE patients suffered from dementia (see **Table 1** for details). According to the information obtained by individual histories, two thirds had an acute onset with meningitis and cranial nerve deficits, which subsided during the course of the disease. A slow but clear-cut progression within the chronic phase was documented in 88% of patients. The disease progression showed stable phases in 50% of these patients, seemed to slow down in 38%, but it was never accelerating in the patients seen.

All patients were native Yakuts (Evenks) as one of the indigenous peoples of the Siberian North. Most patients originated from the Viliuiski river basin north of the Viliuiski river, which is the core region of VE occurrence (**Figure 1**) [3], [7]. Only three of the VE patients were native of the central Yakutsian region surrounding the capital Yakutsk. The remaining Yakutsian subjects are native from similar regions within the Republic of Sakha.

### Blood cell counts

Retrospective analysis of blood hematology data from patients records of the chronic VE patients and controls revealed that 65% (13/20) of the VE patients had mild to moderate transient elevations of eosinophils (5–22%; normal range: <5%) in the acute/subacute phase of their disease course compared to none of the controls (*P* value: 0.0025 [Fisher's exact test]; Relative risk [95% CI]: 2.14 [1.25–3.68]).

### Clinical electrophysiology

Neurography and myography studies revealed no evidence of motor neuron dysfunction or myopathy, but axonal peripheral neuropathy in one VE patient (7%). Sensory-evoked potentials revealed minor, but definite changes in 7 out of 10 patients (70%) from the lower limbs (N. tibialis), but normal results in all patients from the upper limbs (N. medianus). These results suggest that minor dorsal column dysfunction is frequently observed in VE patients, but no controlled data were available for healthy Yakutian subjects.

### CSF results

Standard CSF studies in the 10 VE patients exhibited a very mild pleocytosis with maximal leukocyte count of 9 cells/mm^3^ in only 3 patients, but normal results in the remaining patients as well as controls (**Table 2**). Although there are more patients with oligoclonal IgG bands (OCIBs; 70% in VE vs. 38% in controls) or elevated β-trace protein content in the CSF (56% in VE vs. 11% in controls), we found no significant differences of CSF parameters studied between the chronic VE and the control group. 33% of VE patients had pathological leukocyte counts on the CSF (mild lympho-monocytic pleocytosis, <10/µL; not significant compared to control group).

**Table 2:** CSF results in VE patients and controls.

Parameter	Unit	Normal range	Number of subjects with pathological results (%)	
			VE patients (n = 10)	Yakutian controls (n = 8)
Total protein (mean±SD [range])	mg/l	<450	341±135 (177–632)	288±124 (121–492)
Pathological total protein levels			2 (20%)	1 of 8 (13%)
Leukocyte count	n/µl	<5	3.8±2.6 (1–9)	2.4±1.1 (2–4)
Pathological CSF leukocyte count			3 (33%)	0 (0%)
OCIBs in CSF	n	<2	7 (70%)	3 (38%)
Albumin quotient		<8×10^−3^	0 (0%)	0 (0%)
Intrathecal IgG synthesis	%	0%	0 (0%)	1 (13%)
Intrathecal IgA synthesis	%	0%	0 (0%)	0 (0%)
Intrathecal IgMG synthesis	%	0%	0 (0%)	0 (0%)
Measles-AI		<1.4	0 (0%)	0 (0%)
Rubella-AI		<1.4	0 (0%)	0 (0%)
Varicella Zoster Virus-AI		<1.4	5 (50%)	4 (50%)
HSV 1-AI		<1.4	4 (40%)	3 (38%)
HSV 2-AI		<1.4	4 (40%)	5 (63%)
FSME-AI (Tick-borne Encephalitis)		<1.4	0 (0%)	0 (0%)
CMV-AI		<1.4	0 (0%)	0 (0%)
LCMV (immunfluorescence assay)		negative	0 (0%)	0 (0%)
Borrelia burgdorferi-AI		<1.4	0 (0%)	0 (0%)
TPHA		negative	0 (0%)	0 (0%)
ANA		negative	0 (0%)	0 (0%)
B-Trace protein (mean±SD [range])	mg/l	15–30	31±10 (14–48)	23±10 (13–42)
Pathological β-Trace protein levels			5 (50%)	1 (12%)

Data are number of patients (%) with pathological results, or mean±SD (range).

Fisher's exact test for comparison of pathological vs. normal results did not reveal significant differences between both groups for all parameters.

OCIBs, oligoclonal IgG bands; AI, antibody index.

### Immunodiagnostic results

Elevated AI for Varicella zoster virus, HSV1 and HSV2 showed relatively high prevalences ranging between 33–56% without a significant difference between VE patients and controls most likely to be caused by endemic infection rather than specific cause of VE (**Table 2**). Serum and CSF samples were tested negative for antibodies against LCMV. We did not detect antinuclear antibodies.

The eosinophilia during the acute VE disease course prompted us to further analyze a possible involvement of infectious agents associated with eosinophilic meningitis [16], such as various nematode species (*T. canis*, *Angiostrongylus cantonensis*) and LCM Virus. The results are summarized in **Table 3**. There was a significant increased frequency of serum antibodies against *T. canis* in chronic VE patients (70%) compared to controls (13%; *P* = 0.025; Fisher's exact test), but not for *Angiostrongylus cantonensis*. Statistical comparison with another healthy control population from the Republic of Sakha published in 2011 by Magnaval and colleagues [17] using a similar *T. canis* immunoblot detection system revealed also a significant higher frequency of *T. canis* serum antibodies in chronic VE patients compared to controls (4 out of 90 healthy controls [4.4%]; *P*<0.001; Fisher's exact test). The only control patient showing antibody reaction against *Toxocara* was classified as having MS based on clinical (myelopathy) as well as imaging (multiple periventricular lesions) findings. To further assess the specificity of the anti-*Toxocara* results sera from a control group consisting of 9 MS patients from Yakutsk area were analyzed and one additional 1 MS patient showed a weak immunoreaction against *Toxocara*.

**Table 3:** Immunoreactivity against organisms associated with eosinophilic meningitis in patients with VE and controls.

Parameter^a^	Number of subjects with pathological results (%)		*P* values (Relative risk [95% CI]$)
	VE patients (n = 10)	Yakutian controls (n = 8)	
*Toxocara canis* (serum immunoblot)	7 (70%)	1 (13%)	0.025Δ (2.92 [1.09–7.79])
*Toxocara canis* (CSF immunoblot)	0 (0%)	0 (0%)	-
*Angiostrongylus cantonensis* (serum immunoblot)	0 (0%)	0 (0%)	-

$ Relative risks and 95% confidence interval (CI) for comparison of pathological results vs. normal results.

Δ Fisher's exact test for comparison of pathological results vs. normal results.

a Normal results for all tests are: Not detectable.

### Neuroimaging results

**Figure 2:**
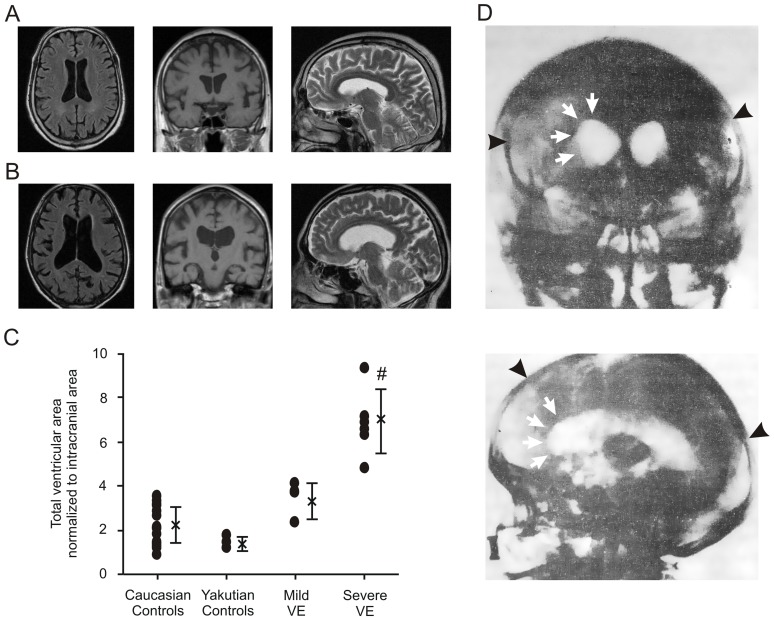
Neuroimaging in chronic Viliuisk encephalomyelitis (VE). (**A,B**) Representative magnetic resonance imaging (MRI) of mild (**A**) and severe (**B**) chronic VE showing severity-dependent enlargement of the lateral and third ventricles. Shown are transaxial FLAIR, coronar T1w and sagittal T2w images demonstrating ventricular enlargement including the 3^rd^ ventricle, periventricular hyperintense signal, thinning of the corpus callosum, but normal cortical and infratentorial structures. The extent of these changes correlated to disease severity. (**C**) Semi-quantitative measurement of ventricular volume in VE patients compared to Yakutian and age- and sex-matched Caucasian controls (see Supporting Information online for technical details). As an estimate of ventricular volumes, the sums of normalized ventricular areas from all slices showing ventricles obtained with a standardized acquisition protocol are displayed (bars and crosses are mean values ± SD). # indicates *P<*0.0001 when compared to all other groups (ANOVA with post-hoc *t*-test including Bonferroni correction). (**D**) Representative pneumoencephalography of subacute VE showing ventricular enlargement (arrows indicate enlarged “bloated” lateral ventricles) and absent air filling of the subarachnoidal spaces of the hemispheric convexities (arrowheads indicate the stops of air filling), suggestive for arachnoideal adhesions.

Neuroimaging studies in 10 chronic VE patients showed marked enlargement of the ventricular system including ventral and dorsal horns of the lateral ventricles and the third and fourth ventricles with signs of CSF diapedesis (**Figure 2** ***A,B***) combined with thinning of the *corpus callosum* in 6 patients (60%) suggestive of hydrocephalus in 9 (90%), and few white matter lesions in 2 (20%), open aqueduct in all cases, and mild cortical atrophy in a subset of cases (60%). Pathological gadolinium enhancement was not observed. To quantify the ventricular size, we performed semi-quantitative analyses of ventricular volume (see Supporting Information). We thereby showed that the degree of hydrocephalic enlargement of the inner ventricular system clearly corresponded to the severity of gait disturbances (**Figure 2** ***A–C*** **; Figure S2**). We classified these VE patients into the categories “mild VE” defined by minor gait apraxia with the ability to walk without assistance (n = 4) and “severe VE” defined as patients who were not able to walk without assistance (n = 6). Spinal MRI was normal in all cases and pathological gadolinium enhancement has never been detected.

The reviewed neuroimaging studies of chronic VE patients previously collected by the VE Institute showed particularly an enlargement of the supratentorial ventricles and were therefore similar to the findings described above for 10 VE patients studied during the expedition.

### Histology

**Figure 3:**
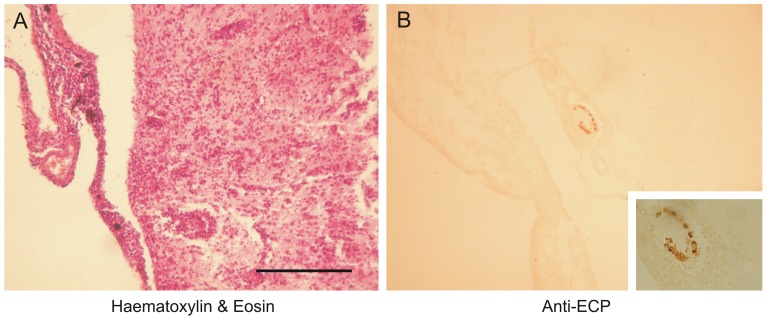
Neuropathology in subacute Viliuisk encephalomyelitis (VE). (**A**) Representative brain histology microphotograph for all three available VE brain samples showed massive intraparenchymal and meningeal infiltrations (Giemsa and DAPI staining confirmed, not shown). (**B**) Anti-ECP immunohistochemistry proved increased appearance of eosinophilic leucocytes. Scale bar, 100 µm.

The investigated cortical brain specimens from three patients suffering from subacute/subchronic VE brain demonstrated massive intraparenchymal and meningeal infiltrations (**Figure 3** **; Figure S3**). Immunohistochemistry staining against ECP showed intracranial eosinophilic infiltrations (**Figure 3** **; Figure S3**). Specificity of anti-ECP staining was confirmed using a specimen suffering from histiocytosis (**Figure S3**).

Due to the serological results suggesting *T. canis* infection, we searched for the parasite in the brain tissue, but we could not detect any parasite (**Figure S3**). As positive control, we investigated mice which were infected using *T. canis* eggs 90 days before sacrifice. Multiple worms were found in the brain of these mice all of them brightly stained by the anti-Tcan antibody (**Figure S3**). It remains unclear whether the non-detection is due the limited quality of the available tissue or whether the parasites were not present any more due to the annealed infection in the subacute/subchronic phase of the disease course. DNA isolation from the paraffin-embedded tissue samples for *T. canis* DNA detection was not possible, most likely due to their old age. The latter explanation is strongly supported by normal MRI scans and the CSF results not showing active inflammation in the CNS in the chronic phase of VE.

## Discussion

In search for the pathophysiology and causative agent of VE, we report clinical, serological and neuroimaging data on chronic VE obtained during two medical expeditions to three villages within the Viliuiski river basin in the Republic of Sakha in 2000 and to the capital Yakutsk in 2006. We note the fact that patient recruitment, clinical investigations and biosample collections were performed in a short period of time in an isolated, rural and remote region of Northeastern Siberia with limited access to advanced medical facilities. We thus focused on a small group of 20 patients suffering from chronic VE with a very precise definition of the clinical phenotype as a pivotal prerequisite for defining underlying pathophysiological processes in complex disorders [9].

The core clinical picture of the examined chronic VE subjects consisted of predominant sensory ataxia, gait apraxia, lower limb spasticity, cognitive impairment and bladder dysfunction. From the medical history, the majority of patients showed an acute meningitis-like phase with cranial nerve involvement prior to the chronic phase, which subsided during the course of the disease. A slow disease progression within the chronic phase was detected almost all patients and showed stable phases in 50% of patients. We did not observe accelerating disease progression in any patient. Our data are in agreement with previous reports [3], [4], [6].

The typical clinical symptomatology and disease course together with the close correlation of symptom severity with the degree of the communicating hydrocephalus detected in cranial MRI strongly supports that pressure by the hydrocephalus as a mechanical factor is the major pathogenic mechanism in chronic VE [18]. This is further supported by results of early pneumoencephalography studies, which was the only available imaging technique until the mid 1990ies in East Siberia: Yakutian colleagues described ventricular enlargement and absent air filling of the subarachnoidal spaces of the hemispheric convexities suggestive for adhesions of the arachnoidea in subacute/subchronic cases of VE [19].

The disease course with stable phases and even remissions also does not support the previous consideration that VE consisted of an acute encephalitic phase followed by a chronic infection provoking an extensive inflammatory response with spongiform regions of the central nervous system [3]–[5]. Indeed, this hypothesis could never be proven in spite of intensive efforts. Consistently, we did not find any evidence for an ongoing inflammatory process in the CSF in chronic VE: The high frequency of intrathecal oligoclonal IgG (OCIBs), but no reactions in the multiple Antibody-Index evaluation (MRZH reaction) and normal cells and protein counts, most likely reflects past infections (immunological scar) and there are no indications of an autoimmune process. Elevated CSF β-trace levels in the VE group might indicate activation of leptomeningeal structures and/or oligodendroglial cells in some patients.

Since most of the patients suffered from a meningitis-like acute phase prior to the progressive chronic phase of the disease [3], it is most likely that an annealed meningeal infection initiates the pathological process leading to the communicating hydrocephalus in chronic VE. This is supported by the CSF results showing a past cerebral infection. Laboratory studies revealed transient eosinophilia during the preceding acute meningitis-like phase prompting to an eosinophilic meningitis. Histological analyses confirmed eosinophilic infiltrations in subacute/subchronic VE brain specimens. This is in contrast to previous reports not showing eosinophilic infiltrations [4], [5]. This discrepancy is most likely related to different techniques used to detect eosinophils, because these reports did not report the use immunohistochemistry with an anti-ECP antibody.

Investigations with regard to organism-specific causes of this immune reaction including various neurotropic viruses (measles, varicella zoster virus, herpes viruses 1 and 2, tick-born encephalitis, cytomegaly virus) and bacteria (*Treponema pallidum*, *Borrelia burgdorferi* ssp. [8]) showed similar results in VE patients compared to controls, which are most likely related to endemic infections rather than a specific cause of VE. The more specific serological analysis of infectious agents associated with eosinophilic meningitis [16], such as the nematode species *T. canis* and *Angiostrongylus cantonensis*, resulted in a significant increased frequency of immune reaction against *T. canis* in chronic VE patients compared to two different control samples [17]. In CSF samples, immunoreactivity against nematode species could not be detected, which is frequently reported for neurotoxocarosis, particularly in patients with sole CNS manifestation [20], [21]. It is noted that there is an intensive cross-reaction of *T. canis* with *T. cati* antigens [13]. Although it was assumed in the past that *T. canis* is the main cause of *Toxocara* spp. related diseases, there is recent evidence that humans can also be infected by *T. cati* larvae, and this infection would similarly elicit visceral and ocular larva migrans syndromes [22]. Our positive results from serological tests on specific *T. canis* antigens do thus not necessarily show that the causative parasite was *T. canis* from dogs or foxes/wolves, but past infections with *T. cati* from domestic cats or other felids might also explain our immunodiagnostic results.

These serological results prompted us to search for *T. canis* larvae in the brain tissue, but we could not detect any signs of persistent *T. canis* infection in the brain samples harvested from subacute to subchronic VE patients. It is however noted that in the literature eosinophilic meningitis or meningo-encephalitis is a rare manifestation of a *Toxocara* ssp. infestation and has been reported only in single patients [21], [23]–[26]. Together, our data provide a first hint that VE might be an endemic representation of the naturally rare cerebral manifestation of *Toxocara* ssp. infection. However, the complete lack of data on the incidence/prevalence of *Toxocara* ssp. in local canidae or felid populations or the prevalence of *Toxocara* infestations in the local normal population warrants future parasitological studies within the Viliuiski area to define *Toxocara* ssp. as the definite causative infectious agent for VE.

Together, our data showed that postinfectious communicating hydrocephalus is the major pathogenetic factor to chronic VE, most likely triggered by eosinophilic meningitis. Thus, the clinical and imaging findings as well as the postinfectious cause of chronic VE are quite different from those of neurological diseases in other geographical isolates, such as kuru, HTLV-1 associated tropical spastic paraparesis or the ALS-PD-dementia complex of Guam. The past eosinophilic reaction in VE might be caused by an already annealed infection with *Toxocara* ssp. and might therefore represent the first hint for an initial cause leading to the development of chronic VE warranting further parasitological explorations within the Viliuiski area. Our data provide a framework for future studies and potential therapeutic interventions for this enigmatic endemic neurological disease potentially spreading in Sakha Republic.

## Supporting Information

Figure S1 **Representative image showing the semi-quantitative estimation procedure of the ventricular area/volume.** The ventricular area is marked in red color, and total intracranial area is marked in yellow. 3V, represents third ventricle, LV, represents lateral ventricle.
(TIF)

Figure S2 **Semi-quantitative measurement of ventricular volume of the lateral ventricles (A) and the third ventricle (B) in VE patients compared to Yakutian and age- and sex-matched Caucasian controls.** Mild VE patients were able to walk without assistance, whereas severe VE patients needed help to walk or were unable to walk at all. As an estimate of ventricular volumes, the sums of normalized ventricular areas from all slices showing ventricles obtained with a standardized acquisition protocol are displayed (bars and crosses are mean values ± SD). # indicates *P*<0.001 when compared to all other groups (ANOVA with post-hoc *t*-test including Bonferroni correction).
(TIF)

Figure S3 **Histology of cortical brain sections of subacute VE patients.** (**A–B**) A specimen from a patient suffering from histiocytosis served as positive control for eosinophilia. (**A**) Haematoxylin/eosin staining or (**B**) anti-ECP immunohistochemistry clearly shows eosinophil leucocytes. In (**C–D**) diffuse brain eosinophilia is seen in VE brain specimens (arrows). (**E–H**) *Toxocara canis* species could not be detected in any brain slice investigated. (**E–F**) As positive control we used mice which had been inoculated with *T. canis* eggs 90 days prior to investigation. In Haematoxylin/eosin stainings, worms could be easily detected throughout the whole brain (**E**). These were brightly stained using a polyclonal antibody against Tcan surface protein (**F**). No worms were found in VE brain specimens (**G**) and no Tcan immunostaining could be detected (**H**). Scale bars, 100 µm.
(TIF)

Table S1 **STrengthening the Reporting of OBservational studies in Epidemiology (STROBE) initiative checklist reporting that the report is according to the STROBE guidelines.**
(DOC)
