# Supplementary figures and images for: Communicating Hydrocephalus Following Eosinophilic Meningitis Is Pathogenic for Chronic Viliuisk Encephalomyelitis in Northeastern Siberia
Source: PLoS One. 2014 Feb 28;9(2):e84670. doi: 10.1371/journal.pone.0084670 (PMC3938403; doi:10.1371/journal.pone.0084670)

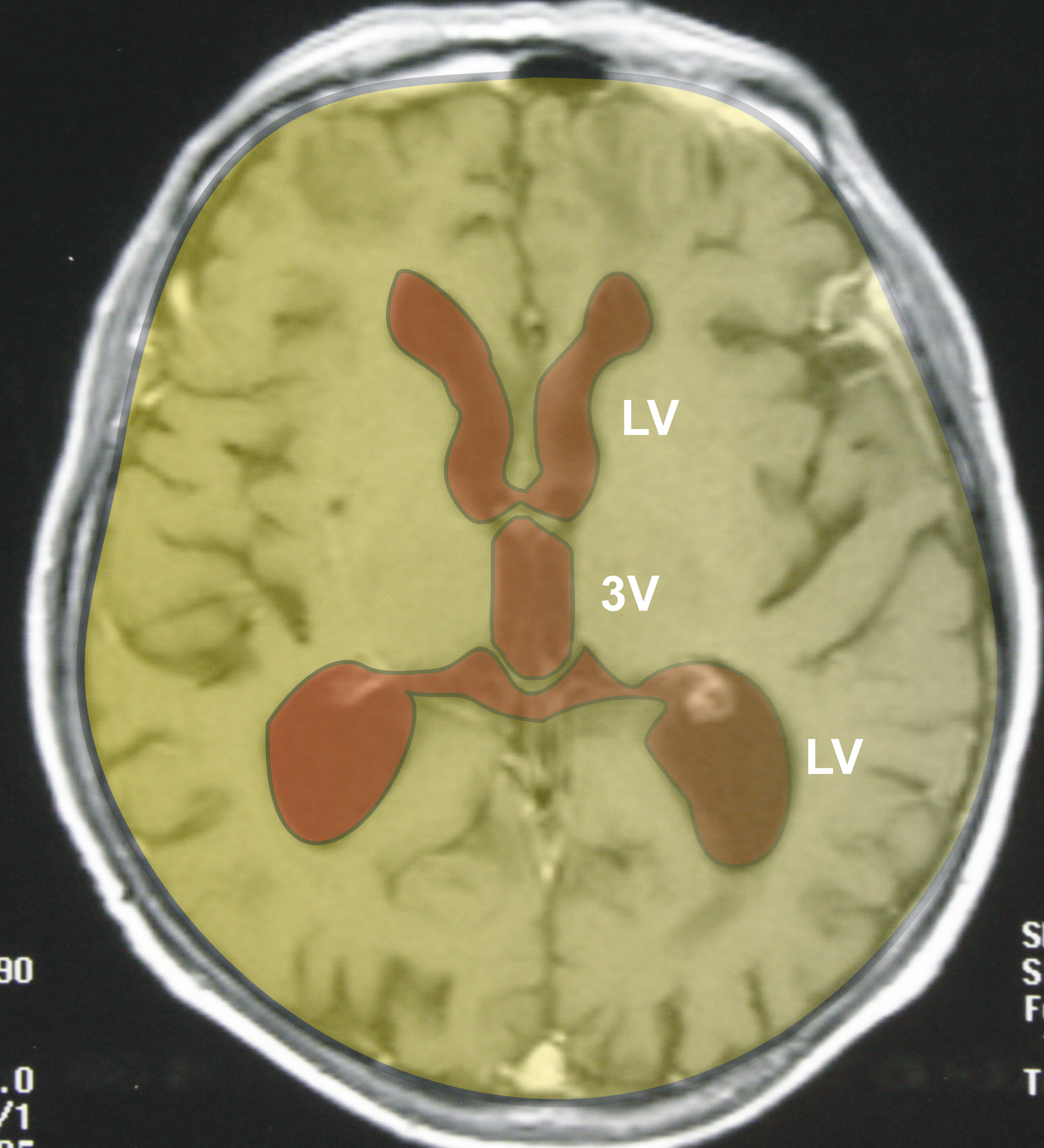

Supplement: Figure S1 — Representative image showing the semi-quantitative estimation procedure of the ventricular area/volume. The ventricular area is marked in red color, and total intracranial area is marked in yellow. 3V, represents third ventricle, LV, represents lateral ventricle. (TIF) [file pone.0084670.s001.tif]

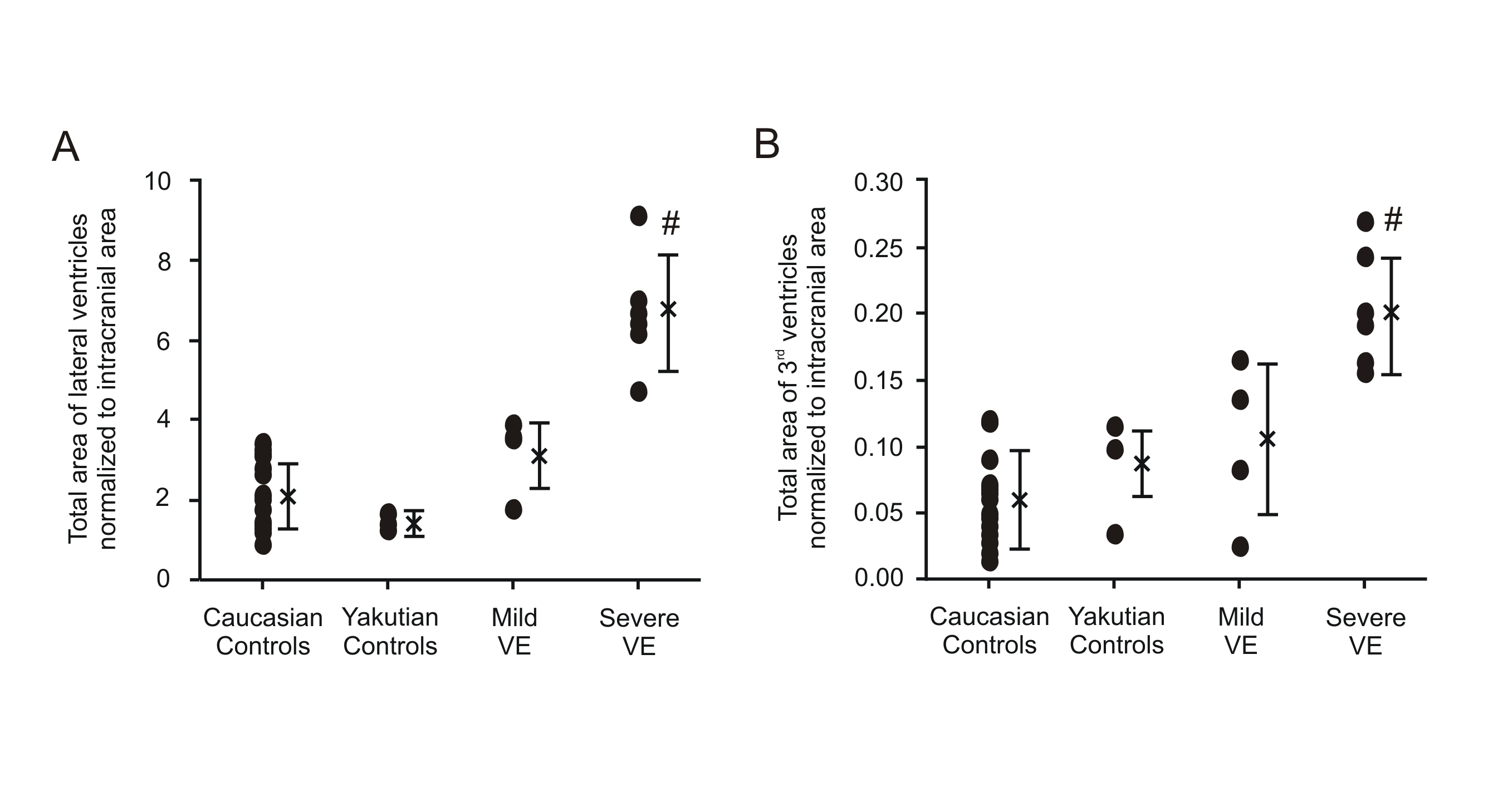

Supplement: Figure S2 — Semi-quantitative measurement of ventricular volume of the lateral ventricles (A) and the third ventricle (B) in VE patients compared to Yakutian and age- and sex-matched Caucasian controls. Mild VE patients were able to walk without assistance, whereas severe VE patients needed help to walk or were unable to walk at all. As an estimate of ventricular volumes, the sums of normalized ventricular areas from all slices showing ventricles obtained with a standardized acquisition protocol are displayed (bars and crosses are mean values ± SD). # indicates P<0.001 when compared to all other groups (ANOVA with post-hoc t-test including Bonferroni correction). (TIF) [file pone.0084670.s002.tif]

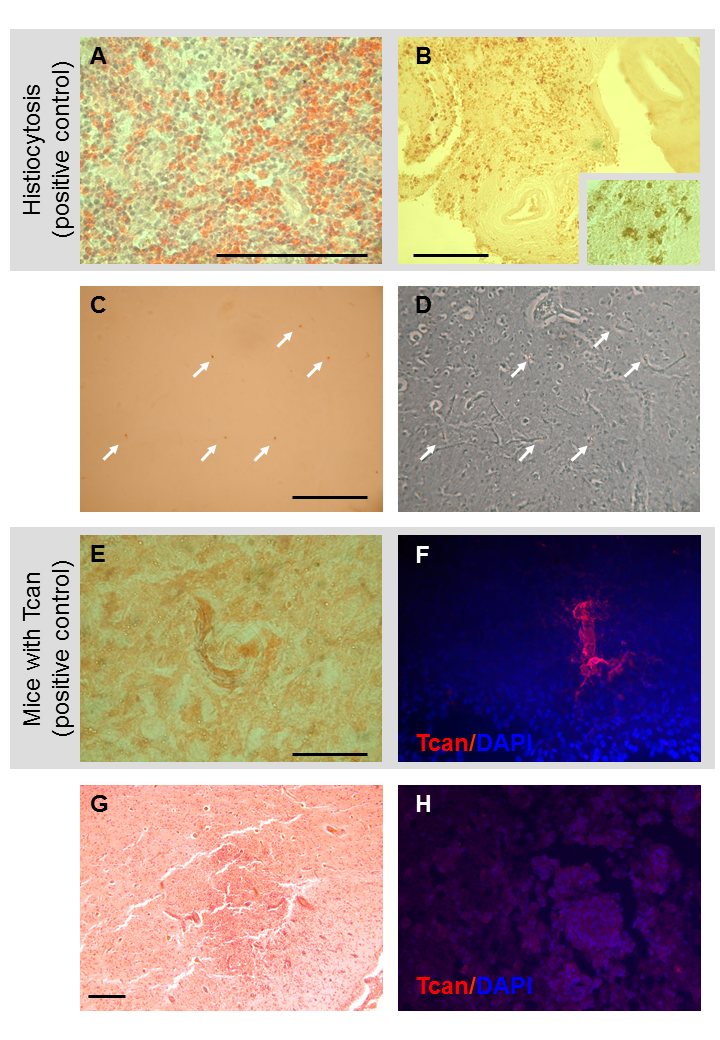

Supplement: Figure S3 — Histology of cortical brain sections of subacute VE patients. (A–B) A specimen from a patient suffering from histiocytosis served as positive control for eosinophilia. (A) Haematoxylin/eosin staining or (B) anti-ECP immunohistochemistry clearly shows eosinophil leucocytes. In (C–D) diffuse brain eosinophilia is seen in VE brain specimens (arrows). (E–H) Toxocara canis species could not be detected in any brain slice investigated. (E–F) As positive control we used mice which had been inoculated with T. canis eggs 90 days prior to investigation. In Haematoxylin/eosin stainings, worms could be easily detected throughout the whole brain (E). These were brightly stained using a polyclonal antibody against Tcan surface protein (F). No worms were found in VE brain specimens (G) and no Tcan immunostaining could be detected (H). Scale bars, 100 µm. (TIF) [file pone.0084670.s003.tif]
